# Danger signals in traumatic hemorrhagic shock and new lines for clinical applications

**DOI:** 10.3389/fphys.2022.999011

**Published:** 2023-01-16

**Authors:** Frédérique Dufour-Gaume, Nadira Frescaline, Venetia Cardona, Nicolas J. Prat

**Affiliations:** ^1^ Institut de Recherche Biomédicale des Armées (IRBA), Bretigny surOrge, France; ^2^ Institut Pasteur, Paris, Île-de-France, France

**Keywords:** traumatic hemorrhagic shock, DAMP, endothelium, platelet, HMGB1, plasma therapy, marker, clinical applications

## Abstract

Hemorrhage is the leading cause of death in severe trauma injuries. When organs or tissues are subjected to prolonged hypoxia, danger signals—known as damage-associated molecular patterns (DAMPs)—are released into the intercellular environment. The endothelium is both the target and a major provider of damage-associated molecular patterns, which are directly involved in immuno-inflammatory dysregulation and the associated tissue suffering. Although damage-associated molecular patterns release begins very early after trauma, this release and its consequences continue beyond the initial treatment. Here we review a few examples of damage-associated molecular patterns to illustrate their pathophysiological roles, with emphasis on emerging therapeutic interventions in the context of severe trauma. Therapeutic intervention administered at precise points during damage-associated molecular patterns release may have beneficial effects by calming the inflammatory storm triggered by traumatic hemorrhagic shock.

## Introduction

Traumatic hemorrhagic shock is the leading cause of death in individuals with war-related injuries. Immediate and early mortality, account for approximatively 60% and 25% of overall trauma-related mortality and late mortality still accounts for 10%–30% of all trauma-related deaths. Most of the late deaths are due to multi-organ failure and infection in a context of Systemic Inflammatory Response Syndrom and secondary immunosupression. A better understanding of the pathophysiology of this condition may aid in the development of novel therapeutic interventions, potentially leading to improved symptom management in patients with combat injuries. Traumatic hemorrhagic shock is not exclusive to military operations, but also occurs in the civilian setting, including road traffic trauma and injuries acquired in armed crimes and terrorist attacks. Death resulting from polytrauma with hemorrhagic shock may either 1) have an early onset and occur due to neurological injury and/or a massive hemorrhage, or 2) have a delayed onset (days to weeks) due to multiple organ failure and sepsis (Oberholzer et al., 2000; Cohen et al., 2009).

When an organ or tissue is subjected to prolonged hypoxia, as occurs with hemorrhagic shock, a switch from aerobic to anaerobic glucose metabolism is activated. This is associated with increased lactate production; disturbances in H^+^, Na^+^, and Ca_2_^+^ ion exchange across the cellular membrane; and mitochondrial dysfunction, which pose severe threats to organ function and homeostasis (Cannistrà et al., 2016). Paradoxically, the restoration of circulatory blood flow and oxygenation to tissue that was previously hypoxic does not immediately improve the lesions caused by ischemia. In fact, reperfusion of ischemic tissues is associated with aggravation and further tissue damage through a phenomenon known as ischemia-reperfusion injury, which is thought to lead to multi-organ failure and death. Within the damaged tissue, necrotic, apoptotic, and stressed cells release multiple molecules into their environment. Danger signals, known as damage-associated molecular patterns (DAMPs) (Relja and Land, 2020), act locally, systemically, or both. Unlike pathogen-associated molecular patterns (PAMPs), DAMPs are endogenous and show extensive range of autocrine, paracrine, and endocrine actions (Skelton and Purcell, 2020).

Throughout this article, a few examples will be described and used to illustrate the pathophysiological roles of DAMPs, with emphasis on emerging therapeutic interventions in the context of severe trauma.

## DAMPs are ubiquitous molecules of diverse nature

The release of DAMPs can be caused by mechanical trauma, ischemia-reperfusion, infection, sepsis, and endotoxemia. Throughout evolution, DAMP-triggered innate and adaptive immune responses have developed to counteract tissue damage, eventually leading to restoration of homeostasis. As an integral part of the innate immune defense mechanism, DAMP actions are designed to protect against sterile injuries, which initially overwhelm the system through a massive release of danger signals, ultimately resulting in hyperinflammation. DAMPs are released by stressed or necrotic cells, as well as by immune cells, activated platelets, and endothelial cells. DAMP activity induces a “hyperactivated” state in a multitude of cells, which further drives inflammatory mediator production and exacerbates the inflammatory response, as seen in severe trauma or sepsis (Fan et al., 2007). In hemorrhagic shock, the whole body is affected by ischemia of hypovolemic origin, although regulatory mechanisms temporarily protect certain organs, such as the brain and heart. Each tissue or organ is a potential source of DAMPs. Although new DAMP molecules remain to be identified, a number of characterized DAMPs are thought to play vital roles in the pathogenesis of severe trauma. These include glycosaminoglycans that line the endothelial wall (syndecans, hyaluronic acid, and heparan sulphate); nuclear proteins, such as High-Mobility Group Box 1 (HMGB1); nucleic acids; coagulation factors, such as S proteins; heat shock proteins (HSP); cytokines; and microvesicles of cellular origin (Table 1).

**TABLE 1:** Damage-associated molecular patterns (DAMPs): physiological activities and pathological activities during traumatic hemorrhagic shock.

Molecule	Normal cellular activity	Actions as DAMPs
**Syndecans**	-Core proteins in the glycocalyx network (Reitsma et al., 2007)	-Degradation of syndecans promotes inflammation and leukocyte tissue migration (Reitsma et al., 2007; Wang et al., 2014)
	-Enzymatic interactions (Reitsma et al., 2007)	-Syndecan-1 level ≥40 ng/ml is a sign of unfavorable outcome (Wang et al., 2014)
	-Keeping platelets and leukocytes towards the center of the vessels (Reitsma et al., 2007)	
**HMGB1 (High-Mobility Group Box 1)**	-Nucleic protein	-Release of cytokines (Bandyopadhyay et al., 2007; Skelton and Purcell, 2020)
	-Protein–DNA binding, transcription, replication, and DNA repair (Bianchi and Agresti, 2005)	-Activation of leukocytes, platelets, and endothelial cells (Tang et al., 2012)
		-Stimulation of platelet aggregation and thrombus formation (Pérez-Casal et al., 2005)
		-Coagulation disturbances: PAI-1, thrombomodulin, tPA, and INR (Cohen et al., 2009)
**S100 proteins**	-Fight against infection, cell division, inflammation, apoptosis, and energy metabolism (Relja and Land, 2020)	-Stimulation of the secretion of E-selectin and vWF by activated endothelial cells (Ueno H, et al., 2004)
		-Increase of apoptotic endothelial cells *in vivo* (Relja and Land, 2020)
		-Increase of vascular permeability (Fiuza C, et al., 2003)
**Nucleic acids**	-Nuclear DNA, RNA, and mitochondrial DNA	-Release of pro-inflammatory cytokines (Dang et al., 2014; Pottecher et al., 2019)
		-Neutrophil extracellular traps (NETs) (Papayannopoulos and Zychlinsky, 2009)
		-Activation of proteases of the cascade coagulation (Shibamiya A, et al., 2007)
**Histones**	-Nucleosomes	-Endothelial cell toxicity (Viemann et al., 2005; Xu et al., 2009)
		-Inhibition of protein C activation (Viemann et al., 2005; Xu et al., 2009)
		-Microthrombi (Viemann et al., 2005; Xu et al., 2009)
		-Increase of thrombin–antithrombin complex (TAT) (Viemann et al., 2005)
**Extracellular vesicles**	-Same composition as membrane of their cellular origin (Liaw PC, et al., 2016)	-Promotion of a procoagulant state of the endothelium (Kannemeier C, et al., 2007)
	-Containing intracellular components (György et al., 2011)	
**Heat shock proteins (HSP)**	-Chaperones for other molecules	-High levels of HSP70 in severe trauma patients (Chabert, 2017)
	-Fight against stresses, including hypo and hyperthermia, UV radiation, and pathogens (Chabert, 2017)	-Ligand of TLR4 (Toll-like receptor) (Chabert, 2017)
		-Correlation between HSP60 and development of acute lung injury after trauma (Kim and Yenari, 2013)

Shown are examples of some molecules known to have DAMP, activity during traumatic hemorrhagic shock. All of these molecules have physiological activities in the absence of trauma. Faced with an attack on the body, high levels of DAMPs, can modify the pro- or anti-inflammatory balance of the systemic response.

**Glycosaminoglycans** line the endothelium wall, forming the glycocalyx network. Under physiological conditions, the glycocalyx moves platelets and leukocytes towards the center of the vessels and promotes numerous enzymatic interactions, e.g., those of the blood clotting cascade. Syndecans are core proteins of the glycosaminoglycans, which are anchored in the endothelial plasma membrane. Syndecan-1 plays an important role in regulating inflammation, and its destruction promotes interaction between endothelium and leukocytes, and the tissue migration of leukocytes (Reitsma et al., 2007).

### HMGB1 (High‐Mobility Group Box 1)

**HMGB1 (High-Mobility Group Box 1)** is a DNA-associated nuclear protein that is not part of the histone family (Bianchi and Agresti, 2005). It facilitates protein–DNA binding, and promotes transcription, replication, and DNA repair. HMGB1 is a ubiquitous protein that is naturally present in its nucleus, and is released into the intercellular space by damaged cells. Certain activated cells can also secrete HMGB1 during stress (Ueno et al., 2004). The human HMGB1 protein consists of two consecutive DNA binding domains (HMG A box and HMG B box) followed by a C-terminal acidic tail and a short N-terminal region. The location of HMGB1 depends on two nuclear localization signals (NLS) and one nuclear export signal (NES). Thus, changes in the NES and NLS can induce modification of HMGB1 location. Also, specific residues in the HMGB1 sequence are responsible for the interaction, binding, activity, and function of HMGB1. Animal studies demonstrated that oxidative stress can induce the release of hyperacetylated HMGB1 (Dave et al., 2009). In addition to acetylation, other modifications, such as methylation, N-glycosylation, phosphorylation, and oxidation, regulate the translocation and release of HMGB1 to the extracellular space in response to various stresses. Depending on the redox conditions of the environment, three redox forms of HMGB1 are associated with modulation of its immunologic functions: 1) intracellular fully reduced HMGB1 can promote the migration of immune cells; 2) partially reduced HMGB1 triggers inflammatory responses and 3) fully oxidized HMGB1 has no chemokine or cytokine activity (Tang et al., 2016).

What is still obscure is how these post-transcriptional modifications are competitively, cooperatively, or independently regulated under trauma conditions.

#### S100 proteins

Are mainly expressed by cells of the myeloid lineage; however, they can also be expressed by epithelial and endothelial cells in an inflammatory situation. This family of proteins has multiple functions, particularly related to fighting infection, cell division, inflammation, apoptosis, and energy metabolism (Relja and Land, 2020). The S100B protein is thought to stimulate the secretion of E-selectin and vWF by activated endothelial cells (Dang et al., 2014).

#### Nucleic acids

Are considered DAMPs because they can reportedly induce the release of pro-inflammatory cytokines (Lee et al., 2011; Pottecher et al., 2019). Activated leukocytes may release nuclear DNA into the plasma, even without cell death. Circulating DNA exists in the form of nucleosomes, which are DNA strands wrapped around their histone chaperones. Nucleosomes are a constituent element of neutrophil extracellular traps (NETs), which are released into the extracellular medium by activated neutrophils (Papayannopoulos and Zychlinsky, 2009). This mechanism is regarded as an essential component of the innate immune response, which is activated following the interaction between HMGB1 and inflammatory cytokines, such as TNF and IL-8. NETs are web-like structures composed of proteins embedded in a scaffold of DNA (chromatin), including both DNA originated from the nucleus and mitochondrial DNA (Granger et al., 2014). On one hand, NETs can attack the plasma membrane of pathogenic microorganisms, but on the other hand, they may have negative effects on the surrounding host tissue—for example, promoting occlusion of the vasculature and thereby causing thrombosis (Granger et al., 2014). A new role of extracellular DNA acting as DAMPS promoting cell necrosis has been demonstrated in several studies (Peer et al., 2016; Jansen et al., 2017). Apart from neutrophils, endothelial cells can also release their DNA into the plasma. In this mechanism, circulating DNA can interact with platelet and other immune cells.

Under physiological conditions, low levels of free DNA are found circulating in the plasma of healthy subjects (Lo et al., 2000). However, high levels of circulating DNA have been demonstrated in many pathological situations, such as sepsis, severe trauma, myocardial infarction, and cancer (Fournie and Courtin, 1995; Chang et al., 2003; Rainer et al., 2003). While any cell death results in the release of nucleic acids into the intercellular medium, their elimination varies depending on whether apoptosis or necrosis has occurred. For example, the elimination of DNA takes 5–10 min when released from an apoptotic cell, compared to several hours when released from a necrotic cell. This difference is probably due to the absence of prior DNA degradation by intracellular nucleases in the event of necrosis (Liaw et al., 2016). Notably, mitochondrial DNA is characterized by the presence of unmethylated CpG motifs, identical to those in bacterial DNA. Mitochondria also express peptides of the N-formyl type, which are similar to bacterial peptides that bind to the receptors on the surface of neutrophils. Thus, bacterial and mitochondrial DNA act on the same neutrophil receptors (Pottecher et al., 2019).

#### Extracellular vesicles

May be categorized into three different groups based on their size and composition: apoptotic bodies, microvesicles, and exosomes. Apoptotic bodies are the largest, with a diameter of 800–5000 nm, and are formed from the plasma membrane of cells. Microvesicles have a diameter of 100–1000 nm, and are plasma membrane fragments produced by activated cells. Finally, exosomes are the smallest extracellular vesicles, with a diameter of 40–100 nm. They are formed from bodies that fuse with the plasma membrane, and release their content into the extracellular environment, thereby participating in intercellular communication among other processes (György et al., 2011; Yamamoto et al., 2016). Vesicles have various cellular origins, including platelets, leukocytes, erythrocytes, and endothelial cells. They are composed of phospholipids and contain nucleic acids, enzymes, and other proteins that play roles in intercellular communication, inflammation, and coagulation (Chabert, 2017).

About 25% of blood-derived EVs are from circulating platelets or platelet precursor cells (i.e., megacaryocytes) which reside in the bone marrow. Platelet-EVs have thrombogenic properties but they also act as cargos for several molecules, including signaling mediators, growth factors, lipids, proteins and nucleic acids. These elements mediate cell-to-cell cooperation, immune reaction, inflammatory response, and reparation (Puhm et al., 2021).

However, the activation process (agonists or mediators, hemodynamic stress … ) mainly affects the final composition and number of Platelet EVs. Milioli et al. revealed for example that EVs from platelets activated *in vitro* by ADP contain different protein in comparison with those activated by collagen or collagen and thrombin (Milioli et al., 2015).

#### Heat shock proteins (HSP)

Are cytosolic proteins that act as chaperones for other molecules. This family of proteins is produced in response to various cellular stresses, including hypothermia and hyperthermia, UV radiation, and pathogens. HSP70 is a well-described HSP, which stimulates both pro- and anti-inflammatory effects through numerous membrane receptors expressed on monocytes/macrophages, microglia, and dendritic cells. Severe trauma increases the expressions of HSP27, HSP70, and HSP90 by perineuronal nets (Kim and Yenari, 2013). Furthermore, Pespeni *et al.* showed significantly higher HSP60 levels in patients with post-traumatic lesions in the lung; however, only one patient presented with direct thoracic trauma (Pespeni et al., 2005).

DAMPs interact with several receptors, including the well-characterized Toll-like receptors (TLRs) and receptor of advanced glycation end products (RAGE).

### DAMP receptors

Toll-like receptors (TLR) are a family of receptors that recognize patterns of pathogens, termed pattern recognition receptors (PRR). Initially thought to be specific for their exogenous ligands (Hunter, 2005), PRRs can actually recognize a wide range of pathogens that are very different in appearance. This may be due to the presence of similar motifs among different organisms, such as bacterial lipopolysaccharide (LPS) or the unmethylated DNA of bacteria and viruses. Moreover, it is thought that PRRs can recognize DAMPs released by sterile tissue damage. TLR-4 recognizes both bacterial toxins (e.g., LPS) and many endogenous ligands (e.g., hyaluronic acid, heparan sulphate, fibrinogen, HMGB1, and HSPs), and can thus be considered to be at the interface between infection and sterile inflammation. TLRs are expressed by innate and adaptive immune cells, as well as by fibroblasts, epithelial cells, and endothelial cells.

Not only TLRs recognize these DAMPs; the RAGE receptor is another important receptor for HMGB1 and S100 proteins (Fan et al., 2007). RAGE is ubiquitous, and shows constitutively higher expression levels in certain tissues, such as the lungs, and in endothelial cells, monocytes, macrophages, and neutrophils (Barbezier et al., 2014). Other important DAMP receptors are the NOD-like receptors pyrin domain containing (NLRPs). These ones are intracellular and can be activated by distinct DAMPs, such as ATP, ROS (reactive Oxygen species), and uric acid. When activated, NLRP1 and NLRP3 lead to the assembly of intracellular multiprotein complexes named inflammasomes. After activation by intracellular DAMPs, inflammasomes allow the caspase-1 dependent cleavage of pro-IL-1β and pro-IL-18 into mature forms (Bortolotti et al., 2018).

DAMPs and their receptors, including HMGB1, and TLRs, are highly conserved in all vertebrates (Fan et al., 2007). HMGB1-like proteins also exist in invertebrates, plants, protozoa, and yeasts and possibly in all eukaryotic cells (Ferrari et al., 1994; Barbezier et al., 2014). Like HMGB1, TLRs are present in mammals and many vertebrates. Very similar structural forms are also found in invertebrates and certain plants. Thus, from an evolutionary perspective, TLRs and DAMPs seem to be among the oldest components of the immune system, and would have appeared even before the separation of the animal and plant kingdoms (Ferrari et al., 1994).

Within the context of DAMP recognition, TLR stimulation depends on cofactors, which are essential and their inhibition blocks TLR activation. DAMPs have very different motives from each other, and likely stimulate TLRs through their different cofactors. It is unclear whether the activation of TLRs by different DAMPs and cofactors leads to a stereotyped or adapted response to different stimulations. The question is further complicated by the finding that the same DAMPs lead to the combined activation of several receptors. Other receptors, such as TLR-2, TLR-9, and RAGE are likely involved, indicating complex signaling pathways, and potential therapeutic pathways (Canaple et al., 1997; Keown-Longo and Higgins, 2017). Finally, the response is affected by the cell type on which receptors are activated by a DAMP. Thus, for each DAMP, three elements must be considered: the cell type, receptor, and associated co-stimulators/inhibitors.

In summary, innate immunity receptors, which are known to be involved in pathogen recognition, also play an important role in DAMPs generation. The available data indicate that these two different responses appear to stimulate the same defense mechanisms. Extensive numbers of DAMPs, receptors, and signaling pathways work tirelessly to restore homeostasis and repair the damage caused by traumatic hemorrhagic shock. However, side effects of this response may become deleterious. Therapeutic intervention administered at a precise point during this response could have beneficial effects by calming the inflammatory storm triggered by traumatic hemorrhagic shock. Extensive knowledge about DAMPs is required to understand their functions in inflammatory responses and restoration of homeostasis.

## Traumatic hemorrhagic shock, DAMPs, and inflammatory response

### DAMPs within the systemic inflammatory response

The pathophysiological course of severe trauma includes a massive inflammatory response from the onset of the trauma. This phenomenon is called systemic inflammatory response syndrome (SIRS), and involves the release of DAMPs (Relja and Land, 2020). In traumatic hemorrhagic shock, sterility is a distinguished feature of SIRS. It clinically mimics sepsis, and contributes to the development of multi-visceral organ failure secondary to severe trauma. SIRS is counterbalanced by an anti-inflammatory compensation syndrome (CARS), which is also modulated by a series of DAMPs, but with anti-inflammatory effects. CARS counterbalances SIRS, but simultaneously plunges the injured person into a state of immunosuppression, which makes a patient susceptible to infections and increases the risk of multi-organ failure (Bandyopadhyay et al., 2007). Chronologically, the onset of CARS is followed by the commencement of SIRS (Relja and Land, 2020; Skelton and Purcell, 2020).

Faced with an attack on the body, DAMPs can be viewed as messengers whose intensity and qualitative composition at any time can modify the interpretation of the message, and the pro- or anti-inflammatory balance of the systemic response. When immune cells are activated by certain DAMPs, such as HMGB1, they release pro-inflammatory cytokines, such as IL-1β, TNFα, IL-6, IL-8, and granulocyte colony-stimulating factor (G-CSF). Anti-inflammatory cytokines also increase IL-10, IL-1 receptor antagonist (IL-1ra), and transforming growth factor-β1 (TGF-β1) (Andersson et al., 2000; Skelton and Purcell, 2020). Macrophages and other activated PBMCs can also actively release HMGB1 (Wang et al., 2001), which makes this protein not only a DAMP but also a cytokine in its own right.

In the same way, concerning inflamasomes. In small quantities, Caspase-1 is a beneficial protein with cell protection function against external stimulation, but when produced in large quantities with inflammasomes in a hyper-inflammatory context, it activates pyroptotic cell death.

In polytraumatized patients, a switch from the Th1 response to Th2 is observed with anergy of the regulatory T cells (Bandyopadhyay et al., 2007; Serve et al., 2018). For example, when monocytes isolated from polytrauma patients are brought into contact with bacterial LPS, the supernatant collected 24 h later shows increased levels of IL-10 (anti-inflammatory), but decreased concentrations of IL-6 and TNFα (pro-inflammatory) compared to controls. This is consistent with the notion of immunosuppression secondary to traumatic hemorrhagic shock (Timmermans et al., 2016). Thus, high levels of both pro- and anti-inflammatory cytokines are critical contributors to the development of multiple organ failure in polytrauma patients, which may be detected within hours after trauma (Bogner et al., 2009).

### DAMPs and lesion kinetics

DAMPs are not released as a single wave but rather in a cascade, with each new release of DAMPs potentially causing a subsequent one. For example, DAMPs released by endothelial cells and activated leukocytes, such as elastase, cytokines, or TNF-α, cause degradation of the glycocalyx that lines the vascular wall. This results in massive release of hyaluronic acid and heparan sulfate into the bloodstream, which, in turn, act as DAMPs. Furthermore, HMGB1 synthesis by monocytes is itself stimulated by TNFα and IL-1, generating a loop of amplification and propagation of the inflammatory response (Bogner et al., 2009).

HMGB1 is released not only in trauma, but also in sepsis and endotoxemia. HMGB1 is a DAMP that is released late in endotoxemia, but early in trauma. In humans, HMGB1 levels in septic shock are correlated with the severity of the condition, and significantly increase from day three post-admission in non-survivors, which is quite late in the disease evolution (Wang et al., 1999). On the other hand, in cases of isolated hemorrhage, the HMGB1 kinetics are much faster, with a plasmatic peak as early as 24 h in murine models of blood loss (Kim et al., 2005). In patients with trauma associated with hemorrhagic shock, the increase of plasma HMGB1 is even earlier, observed from 30 to 60 min following the injury, with a peak occurring between 2 and 6 h (Cohen et al., 2009). Other studies have measured DAMP and cytokine levels in the hours and days following polytrauma (Bogner et al., 2009; Jastrow et al., 2009; Timmermans et al., 2016). Timmermans *et al.* reported significant increases of nDNA, mtDNA, and HSP 70 as soon as treatment was administered at the site of the accident, i.e., less than 30–45 min after the injury. IL-6, IL-8, and the anti-inflammatory cytokine IL-10 were significantly increased upon arrival at the emergency room, i.e., an average of 1 hour after the injury. Therefore, the release of DAMPs and cytokines is very rapid after the injury, and continues for up to several days, depending on the measured molecules. Furthermore, the levels of cytokines IL-6, IL-8, and IL-10 are directly proportional to the levels of nDNA and HSP70 (Tang et al., 2012). Studies have described the interactions between immune cells and DAMPs during the inflammatory process. Other cells, including endothelial cells, are also thought to interact with DAMPs.

## Vascular endothelium is a target and major source of DAMPs

Molecular and cellular components of whole blood interact with vascular endothelium. Present in all tissues and organs, the endothelial cells that line the innermost layer of blood vessels are both a target and a major source of DAMPs in traumatic hemorrhagic shock.

### DAMPs and endotheliopathy

The endothelium responds to physical, chemical, and humoral changes in its environment. In response to these changes, it can secrete many factors involved in inflammation, hemostasis, vasomotion, and vascular permeability. These actions involve certain TLR and RAGE receptors (Cohen et al., 2009; Relja and Land, 2020). Through the endothelial cell RAGE receptor, HMGB1 and S proteins participate in the activation and endothelial secretion of pro-inflammatory cytokines, such as S100B, TNFα, IL-8, and monocyte chemotactic protein-1 (MCP-1) (Relja and Land, 2020). They also enable the phenomenon of immune cell extravasation at the origin of tissue inflammation. Activated endothelial cells increase expressions of the cell adhesion molecules ICAM-1 and VCAM-1, which are necessary for leukocyte margination in tissues, and of RAGE itself (Fiuza et al., 2003). The increased number of RAGE receptors causes a loop of signal amplification and worsening of the endothelial disease. Moreover, endothelial cell hyperexpression of the S100B protein in culture is accompanied by an increased number of apoptotic cells.^4^ Other DAMPs of the S protein family act on endothelial disease by decreasing expression of intercellular adhesion proteins, which increases vascular permeability (Viemann et al., 2005).

DNA circulating in the form of nucleosomes is an important DAMP, capable of inducing cell death. It has been hypothesized that DNA-chaperoning histones are DAMPs that interact with the negative charges on glycosaminoglycans of the endothelial glycocalyx (Xu et al., 2009; Ammollo et al., 2011). Xu *et al.* have demonstrated that extracellular histones are cytotoxic towards endothelial cells, with a dose-dependent effect *in vitro*. Lesions are caused by the entry of calcium into the cell, and are accompanied by increased endothelial permeability, a sign of intercellular dysjunction and suffering. Similarly, *in vivo*, the injection of a high dose of histones into mice causes 100% death within a few hours (Xu et al., 2009; Ammollo et al., 2011). Histologically, the animals present lung lesions with signs of endotheliopathy and its direct complications, including endothelial vacuolation, microthrombi, accumulation of neutrophils in the microvessels, and alveolar hemorrhages. Notable, the authors of this study demonstrated that activated protein C is a powerful inhibitor of histone cytotoxicity. Indeed, this protein, which is involved in fibrinolysis, is also known for its cytoprotective and anti-inflammatory effects. However, histones are also inhibitors of protein C activation *via* endothelial thrombomodulin. Indeed, they essentially bind to the N-terminal domain containing carboxyglutamic acid of protein C, thereby preventing its thrombomodulin-activated cleavage into protein C (Ammollo et al., 2011). Thus, during a traumatic hemorrhagic shock, depending on which component of this fragile balance predominates, the balance can favor a return to endothelial homeostasis or aggravation of the lesions. Free histones are therefore particularly toxic DAMPs. Studies have also shown correlations between lesion severity in polytrauma patients, secondary development of organ failure, and blood levels of extracellular histones, particularly for levels >50 μg/ml (Ammollo et al., 2011; Abrams et al., 2013).

### DAMPs and coagulopathy

Certain DAMPs can have actions affecting molecules involved in the coagulation cascade and its regulatory mechanisms. Notably, in addition to their inhibitory action on protein C, histones can trigger increased thrombin–antithrombin levels and the formation of pulmonary microthrombi (Xu et al., 2009). At the same time, high HMGB1 levels are associated with disturbances of various coagulation proteins, such as PAI-1, thrombomodulin, tPA and, finally, the INR, which increases in parallel to HMGB1 (Cohen et al., 2009). Furthermore, high S100B protein levels are associated with those of vWF secreted by endothelial cells. Studies show that circulating DNA degradation is controlled by a factor VII-activating protease (FSAP) (Nakazawa et al., 2005; Shibamiya et al., 2007), which exists in plasma in the form of a zymogen, and is activated upon contact with apoptotic or necrotic cells *via* histones, glycosaminoglycans, RNA, and DNA. However, activated FSAP is also blocked by several inhibitors, which are also involved in coagulation and fibrinolysis, including α2-antiplasmin (AP), antithrombin III (AT-III), plasminogen activator inhibitor-1 (PAI-1), and the C1-inhibitor (C1-inh) of complement (Nakazawa et al., 2005; Shibamiya et al., 2007). These molecules are involved in several processes, providing evidence for the linkage between cell death, inflammation, and coagulation. This linkage was confirmed by *in vivo* observation that DNA, and even more so circulating RNA, can activate proteases that, in turn, activate factors XI and XII of the coagulation cascade (Kannemeier et al., 2007). Finally, with regards to microvesicles, in an inflammatory situation, the quantity of phosphatidyl-serine present on their surface is significantly greater than the basal expression of phosphatidyl-serine by the plasma membranes from the same cell types. Phosphatidyl-serine interacts with the endothelium and promotes a procoagulant state, with decreased clotting time and increased activated factor X levels and thrombin generation (Zhang et al., 2016). In contrast, other endothelial microvesicles released after stimulation by C-activated protein diffuse an antithrombotic potential that likely inhibits the amplification of coagulation. Indeed, the activated protein endothelial protein C receptor (C-EPCR) complex is intact and still active on the membrane of the endothelial microvesicles formed in this manner, unlike the vesicles formed *via* metalloproteinases, which partially degrade the EPCR (Pérez-Casal et al., 2005).

### DAMPs and platelets

TLR-4 is a receptor present on the surface of immune cells, as well as platelets. This allows HMGB1 to participate in platelet activation, involving release of their granules, aggregation, and formation of a thrombus. Platelets activated in this manner will themselves release large quantities of HMGB1. In a mouse model of severe trauma, Vogel *et al.* showed that platelets are the main source of HMGB1 in thrombi found in the liver and lungs (Vogel et al., 2015). In this same model, transgenic mice lacking platelet HMGB1 exhibited significantly lower plasma levels of the cytokines TNFα, IL-6, and MCP-1 and transaminases compared to wild-type mice (Vogel et al., 2015). In another set of experiments of this study, the authors examined platelet spreading on collagen and vWF using scanning ion conductance microscopy, with or without the addition of recombinant HMGB1, and found that HMGB1 significantly increased the platelet surface area and the speed of platelet spreading (Vogel et al., 2015). These findings confirm the primordial role of HMGB1 in the platelet response. Other DAMPs, the S100A8 and S100A9 proteins, induce thrombogenic and inflammatory responses of endothelial cells by stimulating the expression of thombospondin 1. Thus, the endothelial cell reaction to the presence of S100A8 and S100A9 results in local platelet adhesion and aggregation.

Even if they are anucleated, platelets are also capable of assembling functional NLRP3 inflammasomes leading to the synthesis of IL-1β that is secreted in the extracellular environment, in soluble form and in EVs. *In vitro* studies of vascular injury or thrombin-induced platelet activation showed that inflammasome-mediated IL-1β secretion activates endothelial cells, with subsequent increase in endothelial permeability, neutrophil adhesion, and endothelial transmigration (Hottz et al., 2015). Beyond dengue virus infection (Hottz et al., 2013), platelets mediate increased endothelium permeability through NLRP3-inflammasome activation. The platelet NLRP3 inflammasome has also been described in the context of various other diseases and has been shown to promote platelet activation, platelet aggregation, and platelet-dependent thrombus formation (Murthy et al., 2017; Vogel et al., 2018).

## Discussion

**FIGURE 1:**
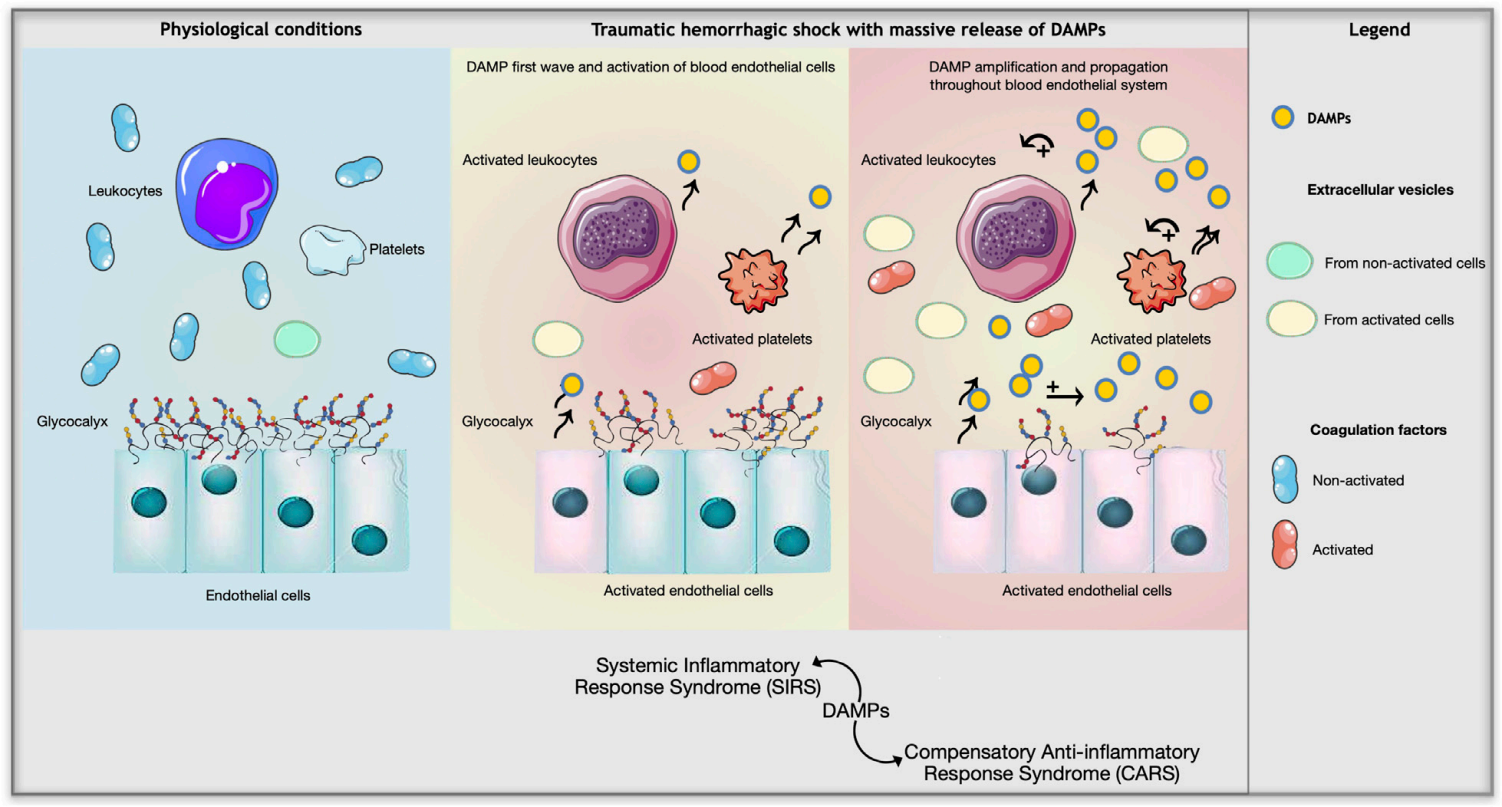
Impact of massive release of DAMPs on the blood endothelial system during traumatic hemorrhagic shock.

The above examples of interactions between DAMPs, the blood–endothelial system, and immuno-inflammatory responses show the complexity of the body’s response to traumatic hemorrhagic shock (Figure 1). Further research is needed to determine how this knowledge can be translated into clinical use for injured patients. For example, we have previously seen that mitochondrial DNA and peptides contain motifs recognized as DAMPs by neutrophil receptors. However, mitochondria are organelles present in all tissues. Thus, a direct traumatic lesion or cellular hypoxia at any location in an organism can cause the real-time release of mitochondrial DAMPs. Additionally, HMGB1 and the S100 proteins are also ubiquitous, and are capable of activating neutrophils, as well as endothelial cells and platelets. The plasma levels of all these DAMPs, and of the pro-inflammatory cytokines and other proteins expressed by the activated endothelium, rapidly increase after hemorrhagic trauma, and thereby participate in activation of the systemic inflammatory response and aggravation of tissue damage. These processes are of interest for several purposes in medical practice: indicating the overall level of seriousness of an injury, marking the specific suffering of certain organs, and potentially being the target of innovative therapeutic methods.

### DAMPs as markers of global severity

In polytraumatized patients, high levels of microvesicles, HMGB1, or S100B are correlated with the injury severity (ISS score), and with a fatal evolution (Cohen et al., 2009; Vogel et al., 2015; Skelton and Purcell, 2020). There is also a proven correlation between plasma levels of HMGB1 and the importance of tissue hypoperfusion and traumatic coagulopathy. Blood levels of HMGB1 are correlated not only with the initial severity of the injury but also with the secondary development of sepsis or multiorgan failure, particularly pulmonary and renal (Cohen et al., 2009; Wang et al., 2014). The same correlations are found with histones and histone-DNA complexes. It is possible that the measurement of DAMPs in hospitalized injured patients could help in patient care by distinguishing those who present high levels compatible with the development of multi-organ failure. In polytraumatized patients, a syndecan-1 level ≥40 ng/mL has been identified as a sign of unfavorable evolution (Gonzalez Rodriguez et al., 2017). Similarly, a vWF antigen assay is currently used to look for innate or acquired deficits, but could also be used to detect an increased plasma level of vWF, as identified in several studies of hemorrhagic shock (Claus et al., 2010).

### DAMPs as markers of specific tissue damage

Apart from general severity, some DAMPs are also markers of more specific tissue damage. Acute renal failure of ischemic origin has extremely serious consequences, potentially including death of the patient. Blood urea and serum creatinine are functional markers, increases of which reflect (sometimes too late) underlying tissue damage. In recent years, new DAMP markers of tissue damage have been identified. Among these, NGAL is a promising biomarker for patient management. A mouse model of sepsis exhibited overexpression of the *NGAL* gene within the first hours following renal ischemia (Mishra et al., 2003). Han *et al.* further demonstrated that the cellular synthesis of NGAL increased fairly rapidly. Additionally, blocking NGAL synthesis with siRNA causes a worsening of apoptosis. Therefore, this poorly understood DAMP seems to be produced by cells in danger as a means of protection from apoptosis (Kjeldsen, 1993). In 2014, Mishra’s team (Mishra et al., 2003) highlighted that an NGAL assay could be of interest for the management of patients with acute renal suffering. In a mouse model of renal ischemia-reperfusion, they showed a 3-fold increase in the tissue expression of NGAL from 3 h post-ischemia, with a peak exceeding 12-fold at 24 h, and a return to basal values at 72 h. In the event of short-term ischemia (5–20 min), the blood creatinine level remained stable while NGAL expression detectably increased, with an intensity that decreased proportionally to the ischemia duration. Finally, a study of patients with acute renal failure treated in intensive care confirmed the usefulness of measuring this DAMP for evaluating the severity of this specific tissue suffering. On admission, the patients exhibited similar blood urea and creatinine levels, while the blood NGAL level was significantly higher in patients who showed an unfavorable evolution at 3 days. The kinetics of blood NGAL levels were also interesting: patients with a favorable evolution at 3 days saw a decreasing level of this DAMP, while those with an unfavorable evolution exhibited a significantly increased NGAL level at day 3 post-intake (Han et al., 2018; Mahmoodpoor et al., 2018).

The lung is another interesting organ in terms of DAMPs. Lungs comprise several cell types along the airways: pneumocyte, ciliated, basal, goblet, and Clara cells. The Clara cell is a non-ciliated cell located at the level of the pre-alveolar terminal bronchioles, which excretes lipids and proteins that enter the composition of the surfactant. Upon secretion by Clara cells, CC-16 passively diffuses across the alveolocapillary barrier into the systemic bloodstream, following a concentration gradient. It appears to exert anti-inflammatory and immunosuppressive effects. Intratracheal instillation of recombinant CC-16 reduces lung inflammation, and CC-16-deficient mice show increased sensitivity to hyperoxic ventilation or ozone (Leclair et al., 2008). The acute respiratory distress syndrome (ARDS) encountered in severely traumatized patients is characterized by elevated blood levels of several proteins that are secreted by the distal pulmonary epithelium, which significantly increase after alteration of the alveolar–air barrier capillary (Lesur et al., 2006). This is the case for CC-16, and Lesur *et al.* demonstrated that the blood level of CC-16 has a prognostic role in ARDS. In ARDS, an early high serum CC-16 concentration is associated with a prolonged duration of mechanical ventilation, and a greater number of associated organ failures (Lesur et al., 2006).

### DAMPs as actors in pulmonary lesions secondary to trauma

Apart from any pre-existing pulmonary pathology, respiratory distress can develop following direct or indirect pulmonary aggression. This pulmonary attack then causes damage to the blood–pulmonary barrier, with the formation of pulmonary edema characterized by presence in the alveoli of plasmatic fluid, as well as epithelial and inflammatory cells. The causes of this so-called lesional lung are generally physical (closed or open trauma) or chemical (intoxication). Lung injury is a classic complication of hemorrhagic shock, traumatic or not. Indeed, even without any direct pulmonary trauma, hemorrhagic shock itself can damage the blood–pulmonary barrier with the edema and inflammatory reaction characteristic of the injured lung (Ciesla et al., 2005; Nasr and Laidi, 2015). In traumatic hemorrhagic shock, the clinical importance of this pulmonary failure is strongly correlated with the severity of the secondary organ failures. Histological signs associated with post-hemorrhagic lung injury include neutrophil infiltration, pulmonary edema, vascular microthrombi, and tissue hemorrhages (Martin et al., 1968). Apart from ischemic lesions and complications directly related to patient resuscitation, several studies reveal that DAMPs are a strong link between hemorrhage and lung injury.

HMGB1 is naturally present in small quantities in the airways. Experimentally, intratracheal instillation of HMGB1 is accompanied by tissue damage with localized hemorrhagic signs and neutrophil infiltration. Notably, the clinical importance of the HMGB1-associated lesions observed in animals is dose-dependent. Such lesions were observed with HMGB1 values greater than the levels found in broncho-alveolar liquid (BAL) in the physiological state (Ueno et al., 2004).

In ARDS, during the final stage of the lesional lung, HMGB1 (which is usually absent from the blood circulation) appears in the plasma with concentrations reflecting the severity of the lesion. Kim *et al* (Kim et al., 2005) showed that HMGB1 is an effector of lesional lung secondary to hemorrhagic shock. In their mouse model, immunohistological analyses show that HMGB1 is secreted by endothelial cells and pulmonary macrophages, as well as by neutrophils that have infiltrated the tissue. In terms of kinetics, the pulmonary level of HMGB1 increases from the fourth hour post-hemorrhage, when the plasma level has not yet increased. Therefore, chronologically, the increase occurs first in the lungs and then in the blood. The same is true for the pulmonary cytokines IL-1β and IL-6. In this model, administration of anti-HMGB1 antibodies 1 hour after hemorrhage strongly reduces the histological signs of lesioned lung, and the local levels of pro-inflammatory cytokines. Similarly, neutropenic mice do not show increased HMGB1 in the lung. Furthermore, while pulmonary cytokines returned to normal values at 24 h, HMGB1 remains elevated up to 72 h post-trauma. Notably, the lung lesions also continue to worsen beyond 4 h post-hemorrhage. These findings are consistent with observations in sepsis, where HMGB1 levels remain elevated above those of pro-inflammatory cytokines, and are correlated with worsening of the patient’s clinical condition. Thus, as a DAMP, HMGB1 is not only a passive marker of lung injury but also a deleterious effector.

HMGB1 is not the only DAMP implicated in post-traumatic ARDS. Animal studies also report that after hemorrhagic shock, NLRP3 inflammasome activation in lung endothelial cells and alveolar macrophages contributes to endothelial damage associated with vascular leakage, edema, increased leukocyte infiltration, and cytokine release in the lungs. Inhibition of the NLRP3 inflammasome attenuates acute lung injury (Yang et al., 2016). In all cases, in both animal models and patients, the lungs are the most sensitive organ—showing earlier and more significant signs of suffering than the kidney or the liver (Ciesla et al., 2005; Pespeni et al., 2005; Abrams et al., 2013).

### DAMP and therapeutic interventions

DAMPs clearly have the potential to be useful markers of injury severity or of patients’ clinical evolution. They are also actors in the complications of trauma hemorrhage, and thus should be included in the biological monitoring of injured patients. In the management of hemorrhagic shock combat-related injuries, the emergency is the etiological treatment of hemorrhage, most often by surgical hemostasis. This is accompanied by measures of vascular filling, regulation of the average arterial pressure, O_2_ supply, and fighting against the phenomena of coagulopathy (Evans et al., 2001; Schlotman et al., 2019). Despite numerous studies of the pathophysiological mechanisms involving DAMPs, and the dysregulation of immuno-inflammatory and endothelial homeostasis, the only current targeted treatment is the use of corticosteroids. These are already applied in the form of hydrocortisone against septic shock with adrenal insufficiency, which reduces hyper-inflammation in these patients without risk of immunosuppression (Annane, 2010). Although the underlying cause is different, septic shock has common elements with hemorrhagic shock. In particular, it is accompanied by hypothalamic-pituitary-adrenal axis dysfunction, which contributes to perpetuation of the inflammatory process due to a lack of cortisol and its anti-inflammatory and immunomodulatory effects. The same abnormalities are present in trauma patients, with initial stress hypercorticism, followed by adrenal insufficiency in half of trauma patients (Hoen et al., 2002). As with septic shock, the HYPOLYTE clinical study has shown that low doses of hydrocortisone can be beneficial in multiple trauma patients with adrenal insufficiency (Roquilly et al., 2011). Apart from hydrocortisone, several studies have tested the use of different immunomodulatory molecules for hemorrhagic shock and sepsis in animal models or even in humans—including granulocyte-macrophage colony-stimulating factor (GM-CSF) and granulocyte colony-stimulating factor (G-CSF), IFN-γ, intravenous immunoglobulins, IL-10, TGF-β, IL-7, Thymosin α1, and others (Thompson et al., 2019). Despite some encouraging results, none of these molecules is now commonly used in the management of traumatic hemorrhagic shock.

Other existing treatments have been tested for their potential protective effect in the management of polytrauma. For example, artesunate is an artemisinin derivative widely used for malaria treatment. In addition to its anti-parasitic activity, it has powerful anti-inflammatory and cell protection effects. In a study of hemorrhagic shock in rats, artesunate-treated animals exhibited a better survival rate, associated with reduced signs of tissue damage (creatinine, transaminases, amyloidosis, CPK, and lactates). This suggests that artesunate acts on the NFκ-B pathway, which is common to DAMPs and cytokines (Sordi et al., 2017). However, it is not currently a recommended treatment in humans for the indication of traumatic hemorrhagic shock.

*Liu et al* showed that the Bruton Tyrosine Kinase (BTK) is a critical regulator of NLRP3 inflammasome activation. Pharmacologic BTK ablation by an inhibitor (ibrutinib) in primary immune cells led to reduced IL-1b, which could be a path for reduction of inflammasome and IL-1b production during trauma hemorrhage (Liu et al., 2017).

The nutrient-rich composition of plasma may make plasma transfusion an effective therapeutic intervention, as its components have the potential to interact with key regulators of inflammation. Among several studies of hemorrhagic shock in severely traumatized patients, the COMBAT and PROMMT studies have shown the benefit of plasma transfusion in association with red blood cells (RCCs), or as a first-line treatment, pending transfusion (Chapman et al., 2015; Wei et al., 2018). Other studies have shown that the administration of glycosaminoglycans—natural components of the endothelial glycocalyx—have a positive effects on its reconstruction *in vitro* and *in vivo* (Zeng et al., 2015; Cannistrà et al., 2016). Similarly, the contribution of albumin seems to limit erosion of the glycocalyx. Other elements of the glycocalyx, endothelial wall, and circulating plasma are also probably involved (Betteridge et al., 2017).

All of these studies have focused on the initial management of hemorrhagic shock. However, the endothelium is both the target and a major provider of DAMPs, and the released DAMPs are directly involved in immuno-inflammatory dysregulation and the associated tissue suffering. Although they are initially released very early after trauma, the release and the consequences of DAMPs continue beyond the initial treatment. Therefore, it seems valid to hope that plasma may have beneficial effects beyond its current acute use. With the use of DAMPs as biological markers of the kinetics of endothelial and immuno-inflammatory disorders and of specific tissue suffering, it could be interesting to test post-surgical plasma administration, during the resuscitation phase of polytraumatized patients.
